# Antioxidant and Anti-Inflammatory Effects of Nettle Polyphenolic Extract: Impact on Human Colon Cells and Cytotoxicity Against Colorectal Adenocarcinoma

**DOI:** 10.3390/molecules29215000

**Published:** 2024-10-22

**Authors:** Magdalena Wójciak, Roman Paduch, Piotr Drozdowski, Weronika Wójciak, Magdalena Żuk, Bartosz J. Płachno, Ireneusz Sowa

**Affiliations:** 1 Department of Analytical Chemistry, Medical University of Lublin, Chodźki 4a, 20-093 Lublin, Poland; weronikawojciak01@gmail.com (W.W.); magdalena.zu25@gmail.com (M.Ż.); i.sowa@umlub.pl (I.S.); 2 Department of Virology and Immunology, Institute of Biological Sciences, Faculty of Biology and Biotechnology, Maria Curie-Skłodowska University, 19 Akademicka Street, 20-033 Lublin, Poland; roman.paduch@mail.umcs.pl; 3 Department of General and Pediatric Ophthalmology, Medical University of Lublin, Chmielna 1, 20-079 Lublin, Poland; 4 Department of Plastic Surgery, Specialist Medical Centre, 57-320 Polanica-Zdrój, Poland; piotr_drozdowski@wp.pl; 5 Department of Plant Cytology and Embryology, Institute of Botany, Faculty of Biology, Jagiellonian University in Kraków, 9 Gronostajowa St., 30-387 Cracow, Poland; bartosz.plachno@uj.edu.pl

**Keywords:** nettle, cytotoxicity, chlorogenic acids, caffeoylmalic acid, interleukins, IL-1β, anti-inflammatory, antioxidant, adenocarcinoma

## Abstract

*Urtica dioica* L. is one of the most widely utilized medicinal plants commonly applied in the form of tea, juice, and dietary supplements. This study aimed to assess the effect of the *U. dioica* ethanol–water extract (UdE) and polyphenolic fraction isolated from the extract (UdF) on normal human colon epithelial cells and to evaluate their protective activity against induced oxidative stress. The cytotoxic potential against human colorectal adenocarcinoma (HT29) and the anti-inflammatory effects were also investigated. UPLC-MS-DAD analysis revealed that both extracts were abundant in caffeic acid derivatives, specifically chlorogenic and caffeoylmalic acids, and therefore, they showed significant protective and ROS scavenging effects in normal human colon epithelial cells. Moreover, they had no negative impact on cell viability and morphology in normal cells and the extracts, particularly UdF, moderately suppressed adenocarcinoma cells. Furthermore, UdF significantly decreased IL-1β levels in HT29 cells. Our research indicates that *U. dioica* may provide significant health advantages because of its antioxidant and anti-inflammatory effects.

## 1. Introduction

Stinging nettle (*Urtica dioica* L.) is one of the most widely utilized medicinal plants, appreciated for its numerous health benefits, lack of toxicity, and widespread accessibility. Traditionally, *U. dioica* was applied in the form of tea, juice, and infusion, but currently, there are also many commercially available dietary supplements derived from nettle [1,2]. Furthermore, in some countries, the plant is used as a food additive and, after cooking or boiling, is added to soups or salads [2,3,4]. It is a rich source of various phytochemicals from the class of secondary metabolites, including terpenes, phenolic acids, flavonoids, chlorophylls, carotenoids, and fatty acids [1,5,6,7,8].

In folk medicine, aqueous and alcoholic preparations from nettle have been used for the treatment of anemia, nasal and menstrual hemorrhage, rheumatism, skin eczema, urinary tract disorders, including gout and bladder and kidney problems, and to enhance lactation [9,10,11]. Recent studies have confirmed the health-promoting value of the plant and evidenced its antibacterial, anti-inflammatory, analgesic, and anti-diabetic properties [12,13,14,15]. Therefore, nettle supplements, including tea, capsules, and leaf powder are gaining increasing popularity and are often used to support overall health, boost the immune system, and alleviate symptoms associated with allergies, such as hay fever. Additionally, nettle supplements are frequently utilized for their anti-inflammatory properties, aiding in the management of conditions like arthritis and promoting urinary tract health, as well as supporting prostate function [16,17].

Furthermore, there are some in vitro investigations indicating the anticancer properties of *U. dioica* extracts against diseases including acute myeloid leukemia (water extract) [18], breast cancer (water and ethanol extract) [19,20], prostate cancer (water extract) [21], and lung cancer (50% methanol extract) [22]. Scientific studies also show the potential of nettle in preventing cancers associated with the gastrointestinal tract and what is important in the context of oral administration of nettle preparations [23]. For example, nettle root water–ethanol extract was found to be cytotoxic against human colon and gastric cancer cell [24]. In turn, the dichloromethane extract from the aerial parts inhibited colorectal cancer cells [25] and the methanol extract from leaves showed cytotoxicity against hepatocarcinoma and colon cancer cells [26]. However, to the best of our knowledge, there are no studies showing the impact of nettle on normal colon cells. Thus, this study aimed to assess the effect of *U. dioica* extracts on the viability of normal human colon epithelial cells and to evaluate their protective activity against induced oxidative stress in these cells. Furthermore, their cytotoxic potential against human colorectal adenocarcinoma and their anti-inflammatory effects in the neoplastic microenvironment were evaluated. The investigated extracts were phytochemically characterized using ultra-high performance liquid chromatography with photodiode array detection and mass spectrometry (UPLC-DAD-MS). The method was chosen for its superior sensitivity and precision in identifying and quantifying plant metabolites compared to other techniques. It is particularly useful for analyzing compounds in complex matrices like plant extracts [27].

Our study focused on polyphenolic components because many biological effects are associated with compounds from this class. They belong to strong antioxidants and anti-inflammatory agents and positively influence cellular health by protecting against oxidative stress and reducing inflammation [28,29]. Nettle is a rich source of polyphenols, mainly from the phenolic acids group, including caffeic acid and chlorogenic acid derivatives [30]. Some flavonoids, such as quercetin, kaempferol, and isorhamnetin glycosides, were also found in the aerial parts of nettle [31]. Most of these compounds are known for their beneficial biological properties [32,33,34].

## 2. Results

### 2.1. Phytochemical Assay of the Extracts

The extract was prepared using ultrasound-assisted extraction (UAE) and 80% ethanol as a solvent, based on previous studies demonstrating the efficiency of this method in extracting bioactive compounds from plant materials. UAE is known for enhancing extraction yields while being time-efficient. In turn, the ethanol–water mixture is effective for isolating a broad range of polar compounds [35,36].

**Figure 1:**
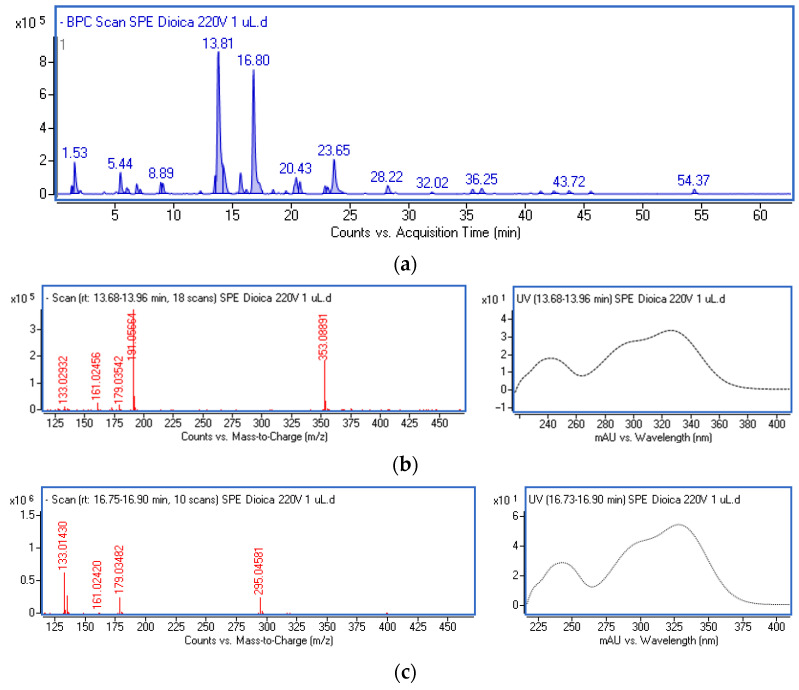
Base peak chromatogram of the polyphenolic fraction isolated from ethanol–water extract of *U. dioica* (**a**); mass and UV-Vis spectrum of chlorogenic acid (**b**); mass and UV-Vis spectrum of caffeoylmalic acid (**c**).

Two extracts were prepared for the investigation: an ethanol–water extract from *U. dioica* (UdE) and an extract obtained by dissolving the polar fraction isolated from nettle (UdF). The phytochemical composition was determined using UV-DAD and MS detection, and the results were calculated per gram of dried extract. From the main peaks visible after chromatographic separation, the UV-Vis and mass spectra were extracted. The identification was carried out using chemical formulas established by the MassHunter software (Version 3.3.2 SP2 build 3.3.2.1037). The identity was further confirmed using commercially available standards or by tentatively identifying the components through comparison of spectral data with the literature [30]. An example of the base peak chromatogram (BPC), along with the mass and UV-Vis spectra of the main components, is shown in Figure 1.

Different caffeic acid derivatives with a fragment ion typical for aglycone (*m*/*z*-H = 179) and a maximum absorption in the region of 322–326 nm were the main phenolic components of the ethanol–water extract from *U. dioica* (UdE) and the polyphenolic fraction (UdF). The main peaks at retention times 13.8 and 16.8 min were identified as chlorogenic acid (*m*/*z* = 353), which is the ester of caffeic and quinic acid, and caffeoylmalic acid (*m*/*z* = 295), respectively. Furthermore, free caffeic acid, four isomers of caffeoylglucaric acid, caffeoylshikimic acid, and crypto- and neochlorogenic acids were found in the extracts. In addition to caffeic acid derivatives, glucoside of dihydroxybenzoic acid, p-coumaroylquinic, p-coumaroylmalic, feruloylquinic, and ferulic acid were also identified. From the flavonoid class, a few quercetin derivatives, including a derivative with *m*/*z* = 609, rutin, quercetin glucoside and acetylglucoside, and kaempferol rutinoside were detected. The detailed chromatographic and mass data, along with the estimated formulas, are included in the Supplementary Materials (Table S1).

Although the polyphenolic profile was similar, significant differences in the quantity of the metabolites were observed between the extract and the fraction. As expected, the number of polyphenols in UdF increased approximately 7–8 times for phenolic acid and approximately 2–3 times for flavonoids compared to UdE as a result of the SPE procedure. In SPE, the matrix was reduced: water-soluble components such as simple carbohydrates were eluted in the prewash step, and hydrophobic components (e.g., fatty acids and chlorophylls) were retained on the SPE column. This is visible when comparing the profiles in the region of 60–90 min on the chromatograms of the extract and the fraction (Figure S1). The predominant components, namely chlorogenic and caffeoylmalic acid, constituted above 85% of the polyphenolic components. Their amounts were 3.17 and 3.86 mg/g in UdE and 22.3 and 33.2 mg/g in UdF, calculated per gram of dried extract. The results of the quantitative analysis are presented in Table 1.

### 2.2. Impact of U. dioica Extracts on Normal Human Colon Epithelial Cells (841 CoTr)

#### 2.2.1. Cells Viability and Cells Morphology

**Figure 2:**
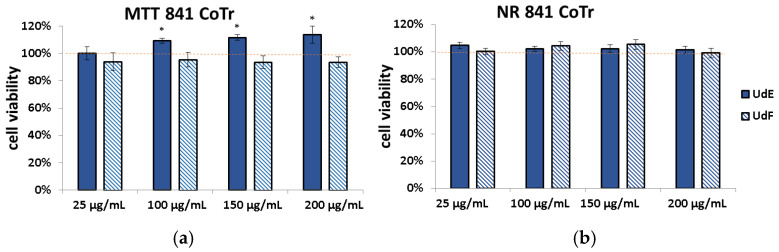
Effect of different concentrations of ethanol extract from *U. dioica* (UdE) and isolated polyphenolic fraction (UdF) on the viability of normal human colon epithelial cells (841 CoTr) assessed by (**a**) MTT and (**b**) NR assays. The results are expressed as a percentage of the control (0.5% DMSO in medium). The red line was set at 100%. * indicates a statistically significant difference at *p* < 0.05 using one-way ANOVA followed by Dunnett’s post hoc test.

To establish the impact of different concentrations of the tested extracts on cell viability, two complementary tests were carried out. The neutral red (NR) test shows the ability of living cells to uptake and accumulate the neutral red dye in lysosomes. The MTT test reflects cell metabolic activity and is based on the conversion of yellow tetrazolium salt by NAD(P)H-dependent cellular oxidoreductase in viable cells to purple formazan crystals. The results are shown in Figure 2.

After 24 h of incubation, it was found that in the concentration range of 25–200 µg/mL, both the ethanol extract from *U. dioica* (UdE) and the polyphenolic fraction (UdF) had no negative impact on the viability and metabolism of normal colon epithelial cells (841 CoTr). Additionally, UdE at concentrations above 100 µg/mL slightly stimulated metabolism by an average of 10% compared to the control (Figure 1a). This indicates a lack of toxicity.

**Figure 3:**
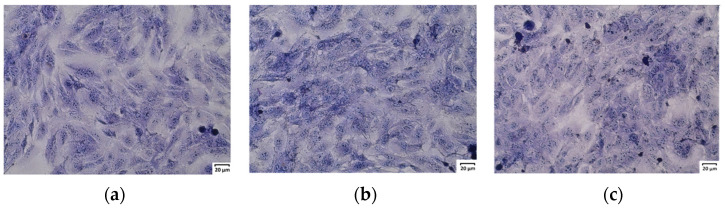
May–Grünwald–Giemsa (MGG) staining of normal human colon epithelial cells: (**a**) control; (**b**) cells after 24 h of treatment with ethanol extract of *U. dioica* at a concentration of 200 µg/mL; (**c**) cells after 24 h of treatment with the phenolic fraction at a concentration of 200 µg/mL. Magnification 100×. Bar = 20 µm.

The observation of the cells after May–Grünwald–Giemsa staining confirmed no negative effect of the extracts on the cells. After 24 h of treatment, the investigated extracts did not affect the morphology of normal human colon epithelial cells. The cells maintained stable contact and no detachment of cells was observed after incubation with the tested extracts. Moreover, normal cells retained the elongated, spindle-shaped characteristic of this culture. Figure 3 presents pictures of 841 CoTr cells treated with 200 µg/mL extract concentration versus the control.

#### 2.2.2. Flow Cytometry Assay

The quantitative analysis of extract-induced cell death was performed using an Annexin V-fluorescein isothiocyanate (FITC)/propidium iodide (PI). The plot of annexin V versus PI was divided into four regions in a clockwise manner. UL represents necrotic cells with annexin V− and PI+; UR represents late-stage apoptotic cells with annexin V+ and PI+; LR represents early apoptotic cells with annexin V+ and PI−; and LL represents healthy cells with annexin V− and PI−.

**Figure 4:**
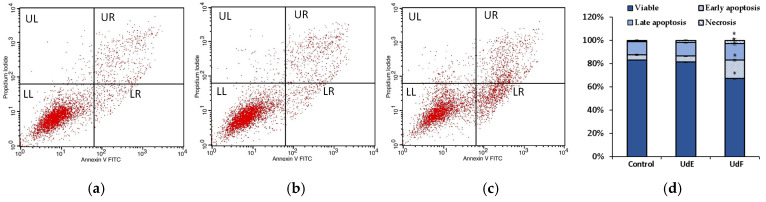
Flow cytometry results obtained in normal human colon epithelial cells (841 CoTr) for (**a**) control; (**b**) after 24 h of treatment with ethanol extract of *U. dioica* at a concentration of 200 µg/mL; (**c**) after 24 h of treatment with the phenolic fraction at a concentration of 200 µg/mL; (**d**) apoptosis rate shown by bar graph. * indicates a statistically significant difference at *p* < 0.05 using one-way ANOVA followed by Dunnett’s post hoc test.

No differences between the control and *U. dioica* ethanol–water extract were noted after 24 h of treatment in the tested concentration range. However, incubation with the polyphenolic fraction at 200 µg/mL caused a decrease in the number of living human intestinal epithelial cells, and the number of cells in late apoptosis and necrotic cells increased compared to the control. No negative effects were observed at lower concentrations of UdF. Flow cytometry results for the control, UdE, and UdF at a concentration of 200 µg/mL are shown in Figure 4.

#### 2.2.3. Cell Cycle

**Figure 5:**
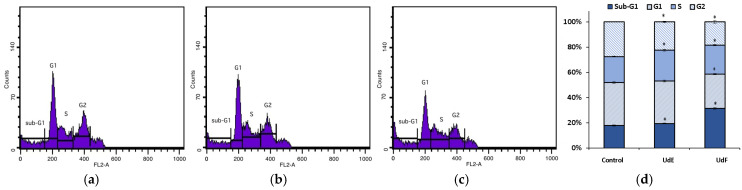
Distribution of normal human colon epithelial cells (841 CoTr) in the particular cell cycle phases: (**a**) control; (**b**) after 24 h of treatment with ethanol–water extract of *U. dioica* at a concentration of 200 µg/mL; (**c**) after 24 h of treatment with the phenolic fraction at a concentration of 200 µg/mL; (**d**) distribution rate shown by bar graph. * indicates a statistically significant difference at *p* < 0.05 using one-way ANOVA followed by Dunnett’s post hoc test.

The influence of extracts on the distribution of cells in the particular phases of the cell cycle, including G0 (a resting stage), G1 (the stage of cell preparation for division), S (the phase of DNA synthesis), and G2 (the stage of genetic material condensation and preparation for division), was further examined. Cell cycle analysis showed that both nettle extracts at the concentration of 200 µg/mL after a 24 h incubation increased the number of normal human colon epithelial cells in the sub-G1 phase of the cell cycle compared to the control. This suggests a probable increase in the number of cells entering apoptosis. The polyphenolic-rich fraction was much more active than the *U. dioica* ethanol–water extract and had a lowering effect on the number of cells in the G1 phase of the cycle (cycle inhibition at the border of the G1 phase). The number of normal cells in the S phase increased after incubation with both extracts, while the number of cells in the G2 phase decreased. This suggests an inhibition of the S phase of the cycle. The results are shown in Figure 5.

#### 2.2.4. Protective and ROS Scavenging Activity

**Figure 6:**
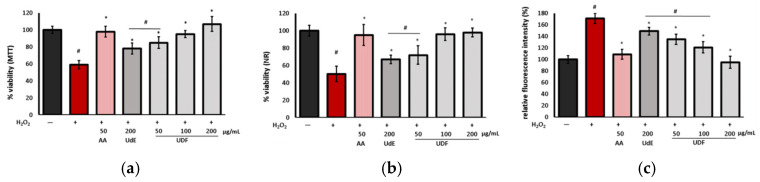
Effect of different concentrations of ethanol extract from *U. dioica* (UdE) and isolated polyphenolic fraction (UdF) on the viability of normal human colon epithelial cells (841 CoTr) in H_2_O_2_-stimulated cells assessed by (**a**) MTT, (**b**) NR assays, and (**c**) relative fluorescence of 2′,7′-dichlorodihydrofluorescein (DCF) in the cells calculated as a percentage in comparison to untreated control cells. The data are means (n = 3) ± SD. * indicates statistically significant difference vs. H_2_O_2_-stimulated cells and # indicates statistically significant difference vs. untreated control. One-way ANOVA followed by Dunnett’s post hoc test was used (*p* < 0.05). AA—ascorbic acid.

The protective effect against oxidative stress and ROS scavenging activity of the extracts were assessed using cell viability assays (NR, MTT) and the fluorogenic H_2_DCFDA, which, after deacetylation in the cells, is oxidized by ROS to fluorescent 2′,7′-dichlorofluorescein (DCF). The results are shown in Figure 6.

The viability of the cells subjected to H_2_O_2_ treatment decreased significantly to 50.2% (NR) and 59.0% (MTT) compared to the control; however, the addition of the extract inhibited the adverse effect of H_2_O_2_. UdE was less active, and only at a concentration of 200 µg/mL, it partially protected the cells against oxidative stress (cell viability was approximately 17–19% higher compared to H_2_O_2_-stimulated cells). In contrast, preincubation of the cells with UdF at 100 and 200 µg/mL maintained cell viability at control levels. These effects were correlated with the impact on ROS production in the cells. UdE effectively reduced ROS at the highest tested concentration, while UdF showed activity at all investigated doses, with the effect being concentration-dependent. At 200 µg/mL of the UdF, ROS levels returned to control levels, and its antioxidant capacity was comparable to that of ascorbic acid.

### 2.3. Impact of U. dioica Extracts on Human Colorectal Adenocarcinoma (HT29)

**Figure 7:**
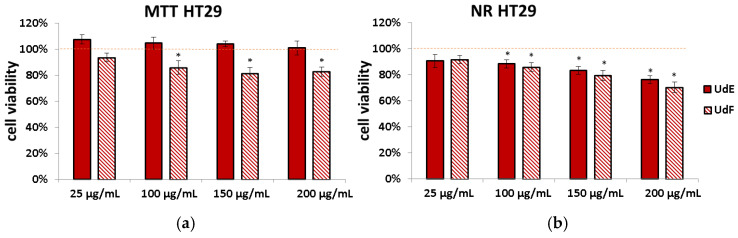
Effect of different concentrations of ethanol–water extract from *U. dioica* (UdE) and isolated polyphenolic fraction (UdF) on the viability of human colorectal adenocarcinoma cells (HT29) determined by the (**a**) MTT and (**b**) NR assays. The results are expressed as a percentage of the control (0.5% DMSO in medium). The red line was set at 100%. * indicates a statistically significant difference at *p* < 0.05 using one-way ANOVA followed by Dunnett’s post hoc test.

The NR assay showed that both nettle extracts reduced the number of viable cells in the HT29 cell line. Moreover, UdF suppressed the activity of NAD(P)H-dependent cellular oxidoreductase (MTT assay) (Figure 7).

**Figure 8:**
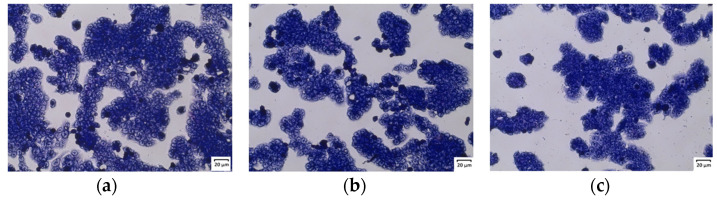
May–Grünwald–Giemsa (MGG) staining of human colorectal adenocarcinoma cells. (**a**) control; (**b**) cells after 24 h of treatment with ethanol–water extract of *U. dioica* at a concentration of 200 µg/mL; (**c**) cells after 24 h of treatment with the phenolic fraction at a concentration of 200 µg/mL. Magnification 100×. Bar = 20 µm.

However, similarly to normal cells, no effect on the morphology of human colon cancer cells was observed. The cells remained in stable assemblies without showing a tendency to loosen (Figure 8).

**Figure 9:**
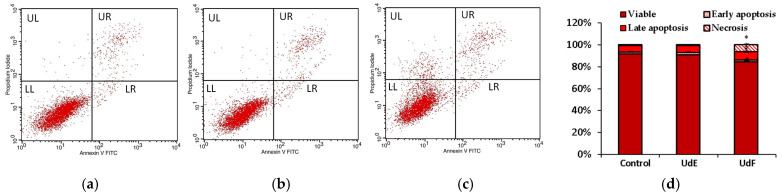
Flow cytometry results obtained in human colorectal adenocarcinoma cells (HT29) for (**a**) control; (**b**) after 24 h of treatment with ethanol–water extract of *U. dioica* at a concentration of 200 µg/mL; (**c**) after 24 h of treatment with the phenolic fraction at a concentration of 200 µg/mL; (**d**) apoptosis rate shown by bar graph. * indicates a statistically significant difference at *p* < 0.05 using one-way ANOVA followed by Dunnett’s post hoc test.

Flow cytometry assay showed a decrease in the number of live HT29 cells by approximately 7% compared to the control after treatment with UdF at a concentration of 200 µg/mL (no effect was observed for lower concentrations). The number of cells in late apoptosis and necrotic cells increased by approximately 2% and 6%, respectively, compared to the control. No statistically significant differences were observed between UdE and the control (Figure 9).

**Figure 10:**
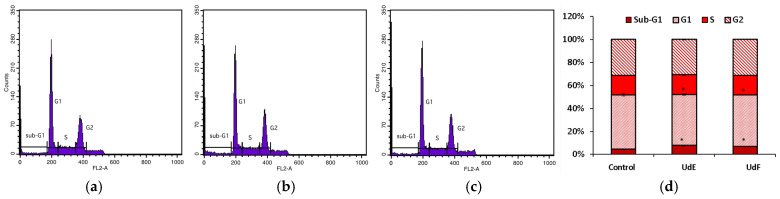
Distribution of human colorectal adenocarcinoma cells (HT29) in the particular cell cycle phases: (**a**) control; (**b**) after 24 h of treatment with ethanol–water extract of *U. dioica* at a concentration of 200 µg/mL; (**c**) after 24 h of treatment with the phenolic fraction at a concentration of 200 µg/mL; (**d**) distribution rate shown by bar graph. * indicates a statistically significant difference at *p* < 0.05 using one-way ANOVA followed by Dunnett’s post hoc test.

Cell cycle analysis showed that both nettle extracts at the concentration of 200 µg/mL after a 24 h incubation significantly increased the population of human colorectal cancer cells in the sub-G1 phase, which may suggest an increase in the number of cells in the initial phase of apoptosis and cycle limitation at the border of the G1 phase (Figure 10). The number of cells in the G1 phase was significantly reduced compared to the control. The number of cells in the S and G2 phase did not change quantitatively after being exposed to the tested extracts compared to the control.

### 2.4. Anti-Inflammatory Activity

Neoplastic changes disrupt the microenvironment and lead to the release of cytokines and chemokines; therefore, anti-inflammatory activity is important in the context of cancer development. As shown in Table 2, UdF at a concentration of 200 µg/mL significantly reduced the release of IL-1β by cancer cells after a 24 h incubation, but none of the tested extracts affected the release of IL-6 and IL-10 in human colon tumor cells.

## 3. Discussion

A lot of studies have shown that *U. dioica* is a valuable plant with a broad range of biological activities and, therefore, may be a promising dietary supplement for complementary or alternative therapy in many disorders [1,2,23]. Various groups of phytochemicals are believed to be responsible for the plant’s activity; however, among them, polyphenols are of particular interest due to their well-established biological effects [37,38]. One of the most desirable features of plant phenolics is their ability to neutralize free radicals because excessive ROS accumulation in cells causes oxidative stress and negatively affects cell structures, leading to lipid and protein oxidation, as well as DNA damage. Compounds with antioxidant activity may support the endogenous antioxidant enzyme system and contribute to reducing the negative effects of oxidative stress [39,40,41].

Our study showed that *U. dioica* (UdE) is a rich source of recognized antioxidants, namely caffeic acid derivatives, and the SPE procedure allowed us to effectively concentrate them while simultaneously reducing the matrix of lipophilic constituents. Both the ethanol–water extract from *U. dioica* (UdE) and the polyphenol-enriched extract (UdF) showed ROS scavenging effects in in vitro H_2_DCFDA assays, and they reduced the ROS production in H_2_O_2_-treated cells. The activity of UdF was significantly higher due to the higher polyphenol concentration, which confirms the leading role of this group of compounds in the antioxidant properties of nettle. The strong antioxidant properties of *U. dioica* have also been demonstrated previously in chemical tests, including ferric reducing/antioxidant power (FRAP), cupric reducing antioxidant capacity (CUPRAC), and free radical scavenging tests (DPPH, ABTS) [14,42,43]. Antioxidant action brings many health benefits, including slowing the aging process, preventing chronic diseases, supporting immune function, and protecting against inflammation [44,45,46]. Therefore, it is a desirable property of dietary supplements and food additives. In this context, *U. dioica* is a valuable plant species that may offer many health advantages.

Further, our investigation showed no negative impact of *U. dioica* extract on normal human colon epithelial cells, as it was not cytotoxic and did not affect cell morphology or the cell cycle. The lack of cytotoxicity of *U. dioica* has also been reported for other normal cell lines including human skin fibroblast and normal bronchial epithelial cells [22,24,42]. The polyphenolic fraction also did not affect cell viability, metabolism, or morphology, as assessed by NR, MTT assays, and microscopic observation, respectively. However, at the highest tested concentration, it increased the number of late apoptotic and necrotic cells and slightly disrupted the cell cycle compared to the control.

While a few studies indicate cytotoxicity of *U. dioica* against gastrointestinal cancer cell lines, our investigation showed only a minor effect of the tested extracts on adenocarcinoma. The extracts, particularly UdF, moderately suppressed the proliferation of cancer cells without impacting cell morphology. Similar to the results observed in normal cells, only slight disruptions in the apoptosis rate and cell cycle were noted. The differences observed compared to the literature data are likely due to varying experimental conditions. For example, Mohammadi et al. found that an extract from the aerial parts of nettle inhibited colorectal cancer, but they used the dichloromethane as the extracting solvent [25]. In their paper, they monitored not only cell viability (MTT assay and trypan blue) and the cell cycle, but also the expression of proteins involved in apoptosis regulation, including Caspase-3, Caspase-9, and Bcl-2. In turn, Kardan et al. used methanol for the extraction of bioactive molecules from nettle and analyzed cell viability (MTT) and the cell cycle [26]. Therefore, it should be noted that the chemical composition of these extracts differed from the ethanol–water extract used in our study, and consequently, the activity of the extracts was different.

Although our study did not show significant anticancer effects of *U. dioica* extracts on HT29 cells, it is worth mentioning that UdF significantly decreased IL-1β, which belongs to the group of pro-inflammatory cytokines that stimulate neoplastic growth. IL-1β mediates many processes occurring in the body. This cytokine connects the intrinsic, oncogene-driven and the extrinsic, inflammatory-disease-driven pathways that link the development of inflammation and the progression of neoplastic disease [47,48]. The level of IL-1β depends on both the stage of cancer development and its primary location. This indicates a number of connections not only with regulation at the level of oncogenes but also with interactions in the tumor microenvironment [49]. This relationship also translates into the dualistic role of IL-1β. It has been shown that it can also exert a protective role and cause the regression of some tumors. It is indicated that the induction of T helper lymphocytes by IL-1β, specifically Th1 and Th17, may play an important role [50]. On the other hand, this cytokine is still an inducer of carcinogenesis, immunosuppression, angiogenesis, and cancer metastases. This may result from the promoting effect of IL-1β on supporting the expression of adhesion molecules, facilitating endothelial permeability, or activation of genes encoding antiapoptotic proteins. In the tumor microenvironment, it significantly affects stromal cells, including TAM macrophages, myeloid suppressor cells (MDSC), Th17 lymphocytes, or B lymphocytes. This increases local inflammation, stimulates tumor progression, and increases drug resistance, as was found in the case of colorectal cancers [51].

IL-1β can induce inflammation through the activation of the NF-κB factor mediated by the IL-1RI receptor. IL-1β affects the disintegration of the NF-κB-IκB complex, leading to the release of NF-κB and its migration from the cytoplasm to the nucleus, where it binds to DNA and activates the transcription of specific genes [52]. IL-1β can also act through the STAT pathway, causing its phosphorylation in a manner dependent on NF-κB [53]. Additionally, the IL-1/JAK/STAT pathway also influences the development of tumor stroma, particularly tumor-associated fibroblasts (CAFs). Limiting the level of IL-1β may have indirect effects related to its influence on the NF-κB pathway and tumor development [54].

Our study showed that nettle polyphenols may exert the potential anticancer effects indirectly through their anti-inflammatory action, as anti-inflammatory activity plays a crucial role in preventing the development of cancer by inhibiting the production of cytokines and growth factors that promote tumor growth. Moreover, reduction of inflammation alleviates the oxidative stress and lowers the levels of reactive oxygen species (ROS), which are responsible for DNA damage and subsequent genetic mutations. Consequently, anti-inflammatory and antioxidant compounds have great significance due to their potential in cancer prevention [55]. Some reports also indicate the anti-inflammatory effects of nettle. It has been found that *U. dioica* extracts inhibit TNF-kappa activation in human T lymphocytes, macrophages, and epithelial cells [56], suppresses cyclooxygenase (COX) and lipoxygenase, which stimulate the pro-inflammatory mediators—prostaglandins and leukotrienes—and decreases the levels of pro-inflammatory cytokines TNF-α and IL-1 [57]. Furthermore, nettle leaf extract decreased IL-1β-induced NF-κB activation and downregulated NF-κB targets, including COX-2 and MMPs, in canine articular chondrocytes [58]. It also reduced NO levels triggered by LPS in macrophages [59]. Also, clinical trials show the anti-inflammatory effect of *U. dioica* in inflammatory bowel disease [60] and rheumatoid arthritis [61].

The biological activity of *U. dioica* extracts is attributed to the abundance of chlorogenic acid (ChA) and caffeic acid (CA) derivatives, which are the predominant polyphenolic constituents of the extracts. Their anti-inflammatory and antioxidant effects have been demonstrated in many studies [62,63]. It has been shown that chlorogenic acid may act directly by reacting with hydroxyl radicals and superoxide anions, as it contains five active hydroxyl groups [64]. Furthermore, it may affect the cellular enzyme system and promote the activation of antioxidant enzymes including superoxide dismutase (SOD), catalase (CAT), and glutathione peroxidase (GSH-Px) [65,66]. It is believed that the Nrf2/ARE pathway (Nuclear Factor Erythroid 2-Related Factor 2/Antioxidant Response Element) is one of the key mechanisms responsible for the antioxidant action of these compounds [67,68,69]. Nrf2 is a redox-sensitive transcription factor that regulates the expression of antioxidant enzymes. During oxidative stress, Nrf2 is released from Keap1, translocates from the cytoplasm to the nucleus, and binds to the ARE in the DNA, triggering the transcription of various antioxidant proteins [70]. Additionally, there are reports that chlorogenic acid activates the Mitogen-Activated Protein Kinases (MAPK) signaling pathway, particularly p38 and ERK1/2, which further modulate the expression of antioxidant enzymes [71]. Activation of antioxidant enzymes plays a crucial role in cancer prevention. By neutralizing ROS, which contributes to damage of cellular components such as DNA, proteins, and lipids, these enzymes help prevent mutations and genomic instability, thereby inhibiting the initiation and progression of cancer. Furthermore, the regulation of ROS levels by antioxidant enzymes can suppress pro-inflammatory signaling pathways, reducing chronic inflammation—a known contributor to cancer development [72].

Anti-inflammatory activity of caffeic acid derivatives is also well documented. For example, it has been found that ChA effectively decreases IL-1β and IL-6 in TNF-α-induced pre-adipocyte 3T3-L1 cells [65], IL-8 in Caco-2 cells stimulated with IFN-γ and myristate [73], and improves symptoms of inflammation in bowel disease [74]. Caffeic acid also shows strong antioxidant effects, as evidenced by chemical tests and cell line assays [75,76,77]. Furthermore, it is able to modulate the expression of mediators of inflammation, including cyclooxygenase, prostaglandins, and interleukins [75,78,79]. Both compounds, in high concentrations, also show a cytotoxic effect on colon cancer cells [76,80] and are likely responsible for the decrease in the viability of colorectal adenocarcinoma cells observed in our study.

In summary, our study shows that *U. dioica* extracts, particularly the polyphenol-enriched extract (UdF), possess strong antioxidant properties and exhibit ROS scavenging effects, which can protect against oxidative stress and related cellular damage. The lack of cytotoxicity on normal human colon epithelial cells further highlights their safety for potential use as dietary supplements or food additives. Although the anticancer effects of *U. dioica* extracts on HT29 cells were not significant, their ability to decrease pro-inflammatory cytokines like IL-1β suggests that they may exert anticancer effects indirectly through anti-inflammatory pathways. Therefore, *U. dioica* could offer potential health benefits due to its antioxidant and anti-inflammatory properties, which are critical in cancer prevention and the mitigation of inflammation-associated chronic diseases.

However, it should be mentioned that in vitro models, though valuable for studying biological effects, lack the complexity of in vivo systems, making it difficult to fully replicate the dynamic interactions that occur within living organisms, such as metabolism, absorption, and tissue distribution [81]. Furthermore, the bioavailability of polyphenols is of great significance to their effectiveness in organisms, as it can vary depending on factors such as their chemical structure, food matrix, and the individual’s gut microbiota [82]. This variability can further complicate the translation of in vitro findings to practical application. As a result, the health benefits of polyphenols observed in vitro may not always be fully realized in vivo. Therefore, further research on the activity of *U. dioica* should focus on investigating its effects on colorectal cancer in animal models. Exploring its synergistic effects with other anticancer agents could also be an interesting direction for future studies.

## 4. Materials and Methods

### 4.1. Reagents and Equipment

Solvents including LC-MS-grade methanol, acetonitrile, and formic acid, and analytical standards were from Merck (Sigma-Aldrich Co., St. Louis, MO, USA). Deionized water was obtained using Ultrapure Milipore DirectQ 3UV-R (Merck KGaA, Darmstadt, Germany). Spectrometric analysis was carried out using a microplate reader (BioTek Instruments, Winooski, VT, USA). FACS Calibur (BD Pharmingen^TM^) with CellQuest Pro Version 6.0 software for the Macintosh operating system (BD Pharmingen™) was used for the flow cytometric assay. Ultra-high performance liquid chromatograph (UPLC) Infinity Series II with a photodiode (DAD) and mass spectrometry (MS) detector (Agilent Technologies, Santa Clara, CA, USA) was used to investigate the polyphenolic composition.

### 4.2. Plant Material and Extracts Preparation

**Figure 11:**
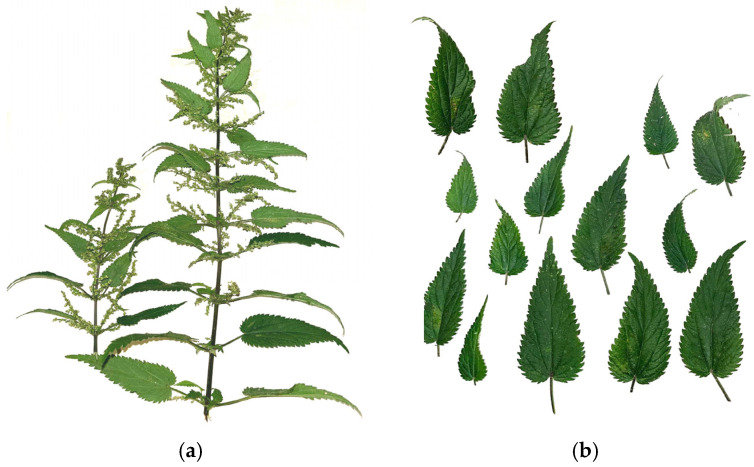
Photographs of (**a**) the collected *Urtica dioica* L. plants and (**b**) the plant part (leaves) taken for investigation.

*Urtica dioica* L. plants were collected in August 2023 from a botanical garden in Lublin (Figure 11). The leaves were washed under running water and freeze-dried (0.001 mbar) for 48 h using a Christ Alpha 2–4 LDplus dryer (Martin Christ Gefriertrocknungsanlagen, GmbH, Osterode am Harz, Germany). The freeze-dried leaves were pulverized and accurately weighed. The extraction of plant material was carried out using 80% ethanol and ultrasound (3 times with a fresh portion of solvent) [83]. The combined extracts were evaporated to dryness (UdE). For the isolation of the polyphenolic-rich fraction (UdF), the obtained extract was subjected to an SPE procedure using LiChrolut^®^ RP-18 SPE tubes (Merck).

### 4.3. Biological Activity Assays

#### 4.3.1. Cell Cultures

The HT29 cell line (ATCC^®^ No. HTB-38™) is a human colorectal adenocarcinoma cell line (grade I), derived from a 44-year-old female adult. The cells were cultured in an RPMI 1640 medium supplemented with 10% fetal calf serum (FCS) (Gibco^TM^, Paisley, UK) and antibiotics (100 U/mL penicillin, 100 g/mL streptomycin, and 0.25 g/mL amphotericin B) (Gibco^TM^, Paisley, UK) at 37 °C in a humidified atmosphere of 95% air with 5% CO_2_. The CCD 841 CoTr cell line (ATCC^®^ No. CRL-1807™) is a normal human colon epithelial cell line (SV40-transformed), derived from a female at 21 weeks of gestation. The cells were cultured in an RPMI 1640 + DMEM (1:1) medium (Sigma-Aldrich) supplemented with 10% FCS and antibiotics at 37 °C in a humidified atmosphere with 5% CO_2_ [84].

#### 4.3.2. Cell Viability Assays

MTT assay: The cells were grown for 24 h in 96-well multiplates in 100 µL of culture medium supplemented with different concentrations of extracts (25–200 µg/mL). Subsequently, the medium was discarded and a new one enriched with MTT solution (5 mg/mL) (25 µL/well) was added. The incubation was conducted for the next 3 h. The formed formazan crystals were solubilized overnight in a 10% sodium dodecyl sulfate in a 0.01 M HCl mixture. The product was quantified spectrophotometrically by absorbance measurement at 570 nm.

Neutral red (NR) uptake assay: Cells were grown for 24 h in 96-well multiplates in 100 μL of culture medium with appropriate concentrations of tested extracts (25–200 µg/mL). Subsequently, the medium was discarded and a 0.4% NR solution medium was added to each well. The plate was incubated for 3 h at 37 °C in a humidified atmosphere with 5% CO_2_. After incubation, the dye-containing medium was removed and the cells were fixed with 1% CaCl_2_ in 4% paraformaldehyde (200 μL). Afterwards, the incorporated dye was solubilized using 1% acetic acetate in a 50% ethanol solution (100 μL). The plates were gently shaken for 20 min. at room temperature, and the absorbance of the extracted dye was measured at 540 nm.

#### 4.3.3. Cytometric Analysis of the Cell Cycle

To examine the influence of extracts on the distribution of cells in the cell cycle phases, the human tumor colon cells (HT29) and normal human colonic epithelial cells (CCD 841 CoTr) were incubated with the tested extracts at a concentration of 200 µg/mL. Floating and adherent cells were harvested, centrifuged (3000 rpm/5 min.), rinsed in PBS w/o Ca^2+^ and Mg^2+^ ions, once again centrifuged, and fixed in 70% ethanol. The control consisted of the untreated cells. The samples were stored for 1 week in −20 °C. Afterwards, the samples were subjected to further steps of the PI staining procedure (PI/RNase Staining Buffer, BD Pharmingen™, BD Biosciences, San Jose, CA, USA). The PI fluorescence intensity was measured using FACS Calibur (BD Pharmingen™), and the obtained data were analyzed using Cell Quest Pro Version 6.0. for the Macintosh operating system (BD Pharmingen™). The results were calculated as a percent of cells in the respective cell cycle phases (sub-G1, G0/G1, S and G2) among all the analyzed cells. In total, 10.000 events were measured per sample.

#### 4.3.4. Flow Cytometry

The Annexin V-fluorescein isothiocyanate (FITC)/propidium iodide (PI) apoptosis kit (BD Biosciences, BD Pharmingen™, San Jose, CA, USA) was used for the flow cytometry method. The HT29 or CCD 841 CoTr cells were seeded into 6-well plates at a density of 1 × 10^5^ cells/well. After cells adhesion (the next day), the growth medium was replaced with a fresh one containing extracts at the concentration of 200 µg/mL. After 24 h of incubation, the floating and adherent cells were harvested, washed with PBS w/o Ca^2+^ and Mg^2+^ ions, and suspended in 1 × binding buffer. The cells were stained with 5 mM of FITC-Annexin V and 5 mM of PI. After 15 min of incubation in the dark at room temperature, the cells were immediately analyzed.

#### 4.3.5. May–Grünwald–Giemsa (MGG) Staining

The cells were incubated in 24-well plates in 1 mL of culture medium supplemented with the tested extracts. After 24 h of incubation (37 °C in a humidified 5% CO_2_/95% air), the medium was discarded and the cell cultures were rinsed with culture medium and stained with the May–Grünwald (MG) stain for 5 min followed by staining for another 5 min in MG diluted in an equal quantity of water. The MG was removed and Giemsa reagent (diluted 1:20 in water) was added to the cells, which were next incubated at room temperature for 15 min. Thereafter, the cells were rinsed three times with water, dried, and subjected to microscopic observations (Olympus, BX51; Olympus, Tokyo, Japan).

#### 4.3.6. Intracellular Levels of Reactive Oxygen Species (ROS)

The cells were grown for 24 h in 96-well multiplates in 100 µL of culture medium supplemented with different concentrations of extracts (25–200 µg/mL). After 30 min of treatment with H_2_O_2_ solution, the cells were incubated with the fluorogenic H_2_DCFDA in the dark for 45 min. The fluorescence of 2′,7′-dichlorofluorescein (DCF) was measured using the excitation wavelength λ = 485 nm and the emission wavelength λ = 530 nm. Ascorbic acid (50 µg/mL) was a positive control [42].

#### 4.3.7. ELISA Assay

The levels of human IL-1β, IL-6, and IL-10 (Elabscience, Houston, TX, USA) were measured immunoenzymatically (ELISA) using commercially available kits according to the manufacturer’s instruction. Briefly, 100 μL of extracts at the concentration of 200 μg/mL were added to the appropriate plate wells. After incubation (2 h) and a series of washings, enzyme-conjugated secondary antibodies (100 μL) were added to the wells and incubated for 1 h. After washing, detection was performed by adding 100 μL of the enzyme substrate to the wells. After 30 min of incubation, the color reaction was stopped by adding 2M H_2_SO_4_ to each well. The optical density of the end product was determined at 450 nm. The concentrations of the cytokines in the analyzed supernatant samples were calculated on the basis of a standard curve. The detection limit was 4.69 pg/mL (IL-1β, IL-6 and IL-10).

### 4.4. Extract Profiling and Quantitative Analysis

Separation of the extracts were carried out using a Titan column (10 cm length, 2.1 mm i.d., 1.9 m particle size) (Supelco, Sigma-Aldrich, Burlington, MA, USA), a mobile phase composed of water (A) and acetonitrile (B), both acidified with 0.1% formic acid at the flow rate of 0.2 mL/min and thermostat temperature of 30 °C. The elution program was as follows: 0–8 min from 98% A to 93% A; 8–15 min from 93% A to 88% A; 15–29 min from 88% A to 85% A; 29–40 min from 85% A to 80% A; and 40–60 min from 80% A to 65% A. DAD and MS working parameters were as previously described [42].

### 4.5. Statistical Analysis

The results are presented as means ± SD from the three experiments. Data were analyzed using one-way ANOVA with Dunnett’s post hoc test. Differences of *p* < 0.05 were considered significant. All tested statistics were at the α = 0.05 level.

## 5. Conclusions

Our study shows that *U. dioica* extracts, particularly the polyphenol-enriched extract (UdF), possess strong antioxidant properties and exhibit ROS scavenging effects, which can protect against oxidative stress and related cellular damage. The lack of cytotoxicity on normal human colon epithelial cells further highlights their safety for potential use as dietary supplements or food additives. Although the anticancer effects of *U. dioica* extracts on HT29 cells were not significant, their ability to decrease pro-inflammatory cytokines like IL-1β suggests that they may exert anticancer effects indirectly through anti-inflammatory pathways. Therefore, *U. dioica* could offer potential health benefits due to its antioxidant and anti-inflammatory properties, which are critical in cancer prevention and the mitigation of inflammation-associated chronic diseases.

To gain deeper insights into the role of nettle extracts, future studies could investigate the induction or suppression of specific proteins involved in various signaling pathways (western blotting). Furthermore, determining the expression of selected genes related to the molecular pathways responsible for metabolic activity (rPCR) could be helpful in understanding the mechanisms underlying nettle’s effects. Additionally, studies on the activity of enzymes involved in maintaining redox balance in the cell, such as superoxide dismutase, catalase, and glutathione, would provide insights into the mechanisms associated with mitigating oxidative stress.

## Figures and Tables

**Table 1:** The results of the quantitative analysis of the main components found in the *U. dioica* extracts expressed as mg/g ± standard deviation of dried extract.

Component	Ethanol–Water Extract (mg/g ± SD)	Polyphenolic Fraction (mg/g ± SD)
Caffeoylglucaric acids (total) ^1^	0.21 ± 0.02	6.35 ± 0.41
Chlorogenic acids (total)	3.17 ± 0.21	22.32 ± 1.11
Caffeoylmalic acid ^1^	3.86 ± 0.24	33.21 ± 1.89
Caffeoylshikimic acid ^1^	0.07 ± 0.00	0.50 ± 0.04
p-Coumaroylmalic ^1^	0.88 ± 0.07	6.20 ± 0.52
Feruloylquinic acids (total) ^1^	0.08 ± 0.01	0.67 ± 0.05
Ferulic acid	0.07 ± 0.00	0.60 ± 0.06
Quercetin derivatives with *m*/*z*-H = 609	0.34 ± 0.02	0.73 ± 0.06
Quercetin acetylglucoside ^1^	0.07 ± 0.01	0.20 ± 0.02

^1^ Quantification based on calibration curve for appropriate aglycone.

**Table 2:** Cytokine concentration (pg/mL) in human colon tumor cells (HT29) after 24 h of incubation with ethanol–water extract from *U. dioica* (UdE) and polyphenolic fraction from *U. dioica* (UdF) at 200 µg/mL concentration.

Cytokine	Control	UdE	UdF
IL-1β	1835.6 ± 109.1	1843.9 ± 127.9	1514.9 ± 96.4 *
IL-6	1677.5 ± 148.5	1670.0 ± 175.2	1735.0 ± 138.2
IL-10	1140.6 ± 117.0	1343.0 ± 124.6	1180.0 ± 142.5

* indicates a statistically significant difference at *p* < 0.05 using one-way ANOVA followed by Dunnett’s post hoc test.

## Data Availability

The data presented in this study are available on request from the corresponding author.

## Supplementary Materials

The following supporting information can be downloaded at: https://www.mdpi.com/article/10.3390/molecules29215000/s1, Table S1: Mass data of the compounds found in the extracts from *Urtica dioica*; Figure S1: Overlapped base peak chromatograms (BPC) of ethanol–water extract of *U. dioica* (red line) and the polyphenolic fraction isolated from ethanol–water extract (blue line).
