# Supplementary material for: Antioxidant and Anti-Inflammatory Effects of Nettle Polyphenolic Extract: Impact on Human Colon Cells and Cytotoxicity Against Colorectal Adenocarcinoma
Source: Molecules. 2024 Oct 22;29(21):5000. doi: 10.3390/molecules29215000 (PMC11547774; doi:10.3390/molecules29215000)
Supplement: Supplementary file 1 [file molecules-29-05000-s001.zip › molecules-3221582-supplementary.pdf]

molecules

MDPI

Article

Antioxidant and Anti-Inflammatory Effects of Nettle Polyphenolic Extract: Impact on Human Colon Cells and Cytotoxicity Against Colorectal Adenocarcinoma.

Magdalena Wójciak [1]*, Roman Paduch [2,3], Piotr Drozdowski [4], Weronika Wójciak [1], Magdalena Żuk [1], Bartosz J. Płachno [5], and Ireneusz Sowa [1]

[1] Department of Analytical Chemistry, Medical University of Lublin, Chodźki 4a, 20-093 Lublin, Poland;
[2] Department of Virology and Immunology, Institute of Biological Sciences, Faculty of Biology and Biotechnology, Maria Curie-Skłodowska University, 19 Akademicka Street, 20-033 Lublin, Poland;
[3] Department of General and Pediatric Ophthalmology, Medical University of Lublin, Chmielna 1, 20-079 Lublin, Poland
[4] Department of Plastic Surgery, Specialist Medical Centre, 57-320 Polanica-Zdrój, Poland;
[5] Department of Plant Cytology and Embryology, Institute of Botany, Faculty of Biology, Jagiellonian University in Kraków, 9 Gronostajowa St., 30-387 Cracow, Poland;

Table S1. Mass data of the compounds found in the extracts from *Urtica dioica*.

R_T (min.)	Mass Data (m/z-H)	Fragment (m/z-H)	Formula	Δ ppm	Component
4.79	315.07261	(152)	$C_{13}H_{16}O_9$	1.44	Dihydroxybenzoic acid hexoside
4.04	371.06231	(209, 135, 179)	$C_{15}H_{16}O_{11}$	0.87	Caffeoylglucaric acid
5.98	371.06167	(209, 135, 179)	$C_{15}H_{16}O_{11}$	−0.85	Caffeoylglucaric acid
6.84	371.06218	(209, 135, 179)	$C_{15}H_{16}O_{11}$	0.62	Caffeoylglucaric acid
8.58	353.08872	(191, 135, 179)	$C_{16}H_{18}O_9$	2.58	Neochlorogenic acid *
8.88	371.06192	(209, 135, 179)	$C_{15}H_{16}O_{11}$	-0.17	Caffeoylglucaric acid
13.78	353.08891	(191, 135, 179)	$C_{16}H_{18}O_9$	3.12	Chlorogenic acid *
15.68	353.08863	(191, 135, 179)	$C_{16}H_{18}O_9$	2.33	Cryptochlorogenic acid *
13.43	179.03551	(135)	$C_9H_8O_4$	2.93	Caffeic acid *
16.80	295.04581	(135, 179)	$C_{13}H_{12}O_8$	-0.44	Caffeoylmalic acid
18.45	497.12909	(135, 179)	$C_{22}H_{26}O_{13}$	-1.96	Caffeic acid derivative
17.38	337.09136	(191, 163, 173)	$C_{16}H_{18}O_8$	-4.53	p-Coumaroylquinic acid (I)
18.73	335.07777	(191, 135, 179)	$C_{16}H_{16}O_8$	1.57	Caffeoylshikimic acid
18.91	337.09253	(191, 163)	$C_{16}H_{18}O_8$	−1.07	p-Coumaroylquinic acid (II)
19.55	367.10304	(191)	$C_{17}H_{20}O_9$	-1.13	Feruloylquinic acid
20.43	279.05133	(133,163)	$C_{13}H_{12}O_7$	1.08	p-Coumaroylmalic acid
23.11	193.05111		$C_{10}H_{10}O_4$	2.46	Ferulic acid *
23.65	531.24299		$C_{25}H_{39}O_{12}$	-3.21	unknown
28.22	609.14785	(300)	$C_{27}H_{30}O_{16}$	2.85	Quercetin derivative
28.87	609.14681	(300, 463)	$C_{27}H_{30}O_{16}$	1.15	Rutin *
29.09	463.08785	(300)	$C_{21}H_{20}O_{12}$	−0.75	Isoquercetin *
32.02	505.09783	(300,463)	$C_{23}H_{22}O_{13}$	-1.85	Quercetin acetylglucoside
33.95	593.14999	(285)	$C_{27}H_{30}O_{15}$	−2.03	Kaempferol rutinoside *
36.25	515.24943		$C_{25}H_{40}O_{11}$	-0.69	unknown
36.30	623.16183		$C_{28}H_{32}O_{16}$	0.11	Unknown flavonoid

* identification was confirmed using standard

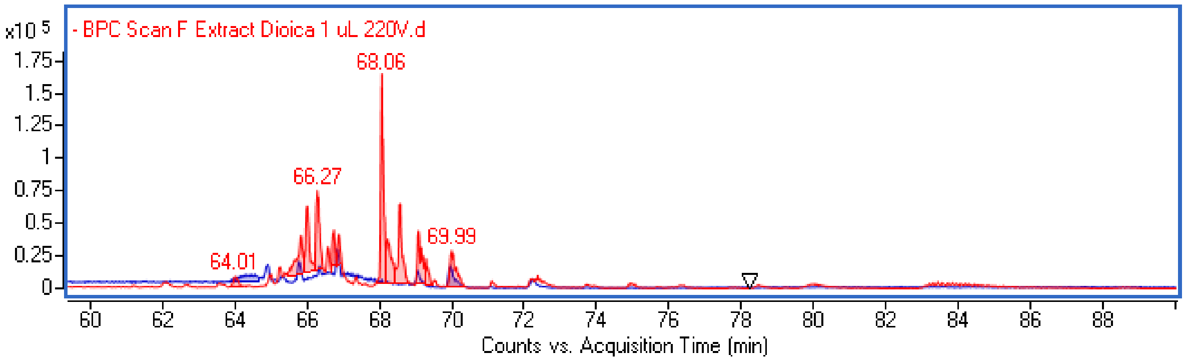

Figure S1. Overlapped base peak chromatograms (BPC) of ethanol-water extract of *U. dioica* (red line) and the polyphenolic fraction isolated from ethanol-water extract (blue line).
